# Efficacy and safety of remimazolam besilate for sedation in outpatients undergoing impacted third molar extraction: a prospective exploratory study

**DOI:** 10.1186/s12903-023-03538-2

**Published:** 2023-10-21

**Authors:** Kana Oue, Aya Oda, Yoshitaka Shimizu, Tamayo Takahashi, Hisanobu Kamio, Utaka Sasaki, Serika Imamura, Eiji Imado, Akari Mukai, Mitsuru Doi, Miyuki Sakuma, Shigehiro Ono, Tomonao Aikawa, Mitsuhiro Yoshida

**Affiliations:** 1 Division of Oral and Maxillofacial Surgery and Oral Medicine, Department of Dental Anesthesiology, Hiroshima University Hospital, Kasumi 1-2-3, Minami-ku, Hiroshima, Japan; 2 Department of Dental Anesthesiology, Graduate School of Biomedical and Health Sciences, Hiroshima University, Kasumi 1-2-3, Minami-ku, Hiroshima, Japan; 3 Department of Oral and Maxillofacial Surgery, Graduate School of Biomedical and Health Sciences, Hiroshima University, Kasumi 1-2-3, Minami-ku, Hiroshima, Japan

**Keywords:** Dental anxiety, Dental fear, Dental procedure, Dental treatment, Remimazolam, Sedation

## Abstract

**Background:**

Dental treatments often cause anxiety, fear, and stress in patients. Intravenous sedation is widely used to alleviate these concerns, and various agents are employed for sedation. However, it is important to find safer and more effective sedation agents, considering the adverse effects associated with current agents. This study aimed to investigate the efficacy and safety of remimazolam besilate (hereinafter called “remimazolam”) and to determine the optimal dosages for sedation in outpatients undergoing dental procedures.

**Methods:**

Thirty-one outpatients aged 18–65 years scheduled for impacted third molar extraction were included in the study. Remimazolam was administered as a single dose of 0.05 mg/kg followed by a continuous infusion at a rate of 0.35 mg/kg/h, with the infusion rate adjusted to maintain a sedation level at a Modified Observer’s Assessment of Alertness/Sedation (MOAA/S) score of 2–4. The primary endpoint was the sedation success rate with remimazolam monotherapy, and the secondary endpoints included induction time, recovery time, time until discharge, remimazolam dose, respiratory and circulatory dynamics, and frequency of adverse events.

**Results:**

The sedation success rate with remimazolam monotherapy was 100%. The remimazolam induction dose was 0.08 (0.07–0.09) mg/kg, and the anesthesia induction time was 3.2 (2.6–3.9) min. The mean infusion rate of remimazolam during the procedure was 0.40 (0.38–0.42) mg/kg/h. The time from the end of remimazolam administration to awakening was 8.0 (6.7–9.3) min, and the time from the end of remimazolam administration to discharge was 14.0 (12.5–15.5) min. There were no significant respiratory or circulatory effects requiring intervention during sedation.

**Conclusions:**

Continuous intravenous administration of remimazolam can achieve optimal sedation levels without significantly affecting respiratory or circulatory dynamics. The study also provided guidance on the appropriate dosage of remimazolam for achieving moderate sedation during dental procedures. Additionally, the study findings suggest that electroencephalogram monitoring can be a reliable indicator of the level of sedation during dental procedural sedation with remimazolam.

**Trial registration:**

The study was registered in the Japan Registry of Clinical Trials (No. jRCTs061220052) on 30/08/2022.

## Background

 Dental treatments often involve unpleasant stimulation, invasive procedures, and pain, which can lead to anxiety, fear, and physical and emotional stress in patients. This stress can result in systemic complications. A recent systematic review estimated that the combined global percentage of adults experiencing anxiety or fear of dental treatment was 31.0%, ranging from mild to severe [1]. Severe anxiety and fear of dental treatment can negatively impact the oral health-related quality of life by hindering patients from receiving necessary dental care and treatment [2, 3]. To mitigate the stress related to dental treatment and complications, intravenous sedation has been widely used [4, 5].

Sedation in dental procedures presents unique challenges for several reasons. First, the treatment site and airway are intricately connected, as dental procedures take place in the mouth where the airway is located. Additionally, a significant amount of water is used in the mouth during dental treatments. Second, the treatment time is relatively lengthy, and most patients are treated as outpatients. Consequently, the drugs and techniques employed for sedation in dental procedures must have broad safety requirements [4]. Sedation in dental procedures necessitates short-acting anesthetics that can achieve rapid onset, easy titration, and rapid recovery from sedation, while still allowing for adjustment to the desired level of sedation. Furthermore, the chosen anesthetic should have minimal inhibitory effects on respiration, circulation, and physiological reflexes. Nonetheless, the perfect anesthetic or technique for dental procedure sedation that meets all these requirements has not yet been established.

Remimazolam besilate (hereinafter called “remimazolam”) is a novel, ultrashort-acting benzodiazepine approved for medical use in the United States, China, and Japan in 2020 and in the European Union in 2021. It has several advantages, including rapid onset of action, short half-life, short recovery time unaffected by dosing duration, minimal effect on respiration and circulation, and low incidence of adverse reactions [6–8].

Based on these pharmacologic characteristics, remimazolam is expected to be safe and effective for a wide range of patients undergoing intravenous sedation for dental procedures [9]. However, there are limited reports examining the efficacy and safety of remimazolam in dental sedation, and the evidence is scarce. This study aimed to investigate the efficacy and safety of remimazolam and to determine the appropriate dosages of remimazolam for sedation in outpatients undergoing dental procedures. This study may contribute to the establishment of safer and more effective sedation management during dental procedures.

## Methods

### Ethics approval and consent to participate

This prospective, single-arm, single-center, open-label clinical trial conducted at Hiroshima University Hospital from August 2022 to March 2023 included outpatients undergoing impacted third molar extraction. The trial was approved by the Ethical Committee for Clinical Research of Hiroshima University and the Hiroshima University Certified Review Board (approval number: CRB2022-0001) and was registered in the Japan Registry of Clinical Trials (No. jRCTs061220052) on 30/08/2022. Following the Declaration of Helsinki and its guidelines, informed consent was obtained from all participants before their study participation.

### Patient inclusion and exclusion criteria

Thirty-one outpatients who were scheduled to undergo impacted third molar extraction were recruited. The inclusion criteria were age between 18 and 65 years old, American Society of Anesthesiologists (ASA) grade I-II classification, and body mass index (BMI) of 18.5 to 30.0 kg/m^2^. The exclusion criteria were patients with a history of heavy drinking, alcohol or drug dependence, those who regularly used benzodiazepines, those with contraindications to benzodiazepines, severe psychiatric disorders, structural brain disorders, abnormal liver or kidney function, and patients who were pregnant.

### Patients

**Fig. 1:**
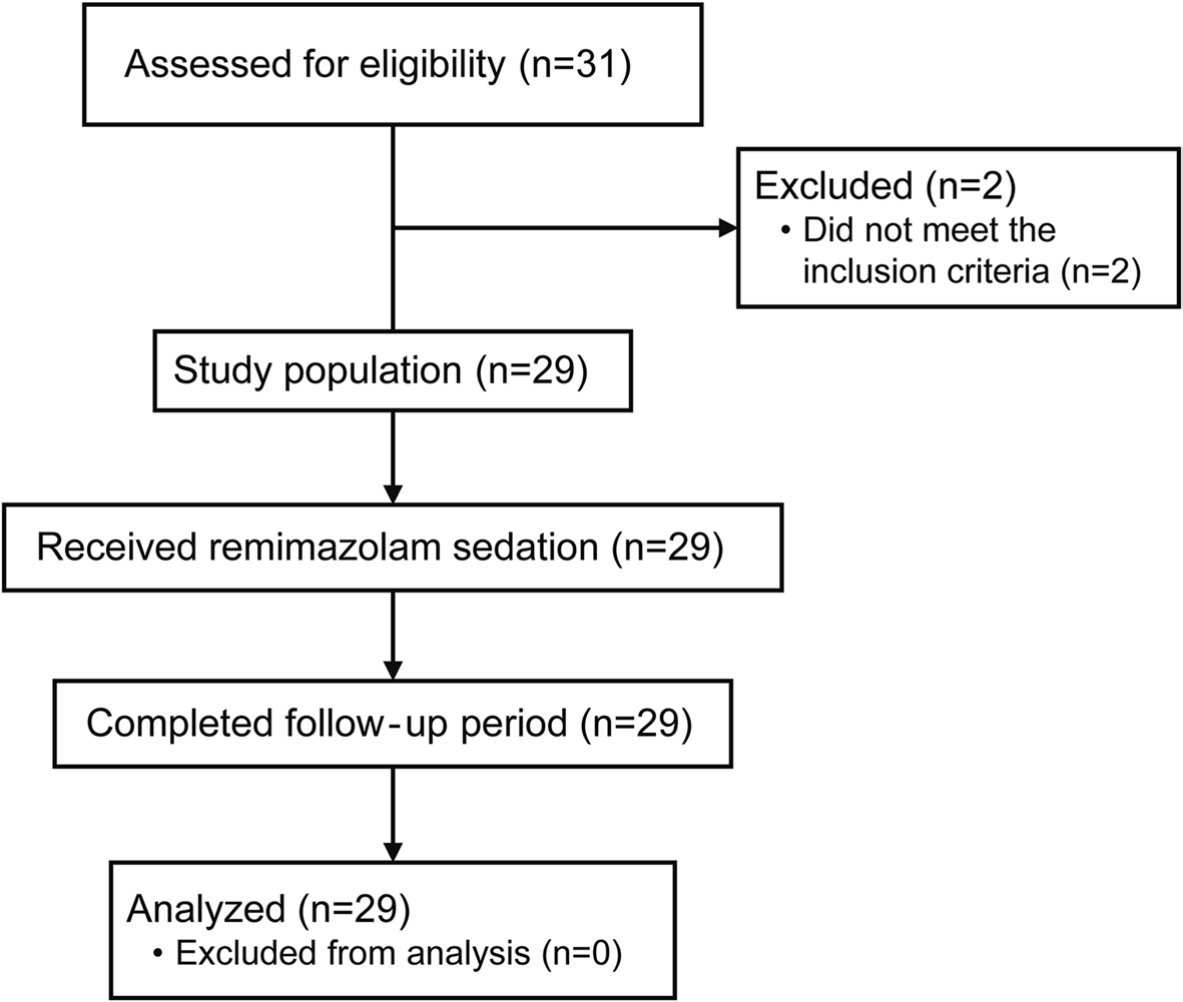
Flow chart of the study design

From August 2022 to March 2023, a total of 31 patients were enrolled in the study. However, two patients were subsequently excluded as they did not meet the inclusion criteria, resulting in a final sample size of 29 patients available for analysis (Fig. 1). The baseline characteristics of the patients enrolled in the study are presented in Table 1. Eight of the participating patients exhibited dental phobia, whereas one patient had a severe gag reflex.

**Table 1:** Demographic and clinical characteristics of the patient population

Age, years	28 (24–37)
Sex, male/female	12/17 (41.4/58.6)
Height, cm	163 (158–169)
Weight, kg	54 (50–60)
BMI, kg/m^2^	20.3 (19.6–21.1)
ASA-PS, I/II	29/0 (100/0)
Dental phobia	8 (27.6)
Severe gag reflex	1 (3.4)

Variables are presented as median (interquartile range) or number of patients (%)

*Abbreviations:ASA-PS* American Society of Anesthesiologists physical status, *BMI* Body mass index

### Study method

Sedation management during dental procedures was the responsibility of a dentist with adequate education and training in anesthesia management, separate from the dentist performing the extraction procedure.

Prior to the procedure, patients underwent fasting for 6 h and were restricted to clear fluids for 2 h. After entering the outpatient dental procedure room, the patients were connected to a monitor (CARESCAPE™ B850, GE Healthcare, Chicago, IL, USA) for continuous monitoring of electrocardiogram (ECG), heart rate (HR), percutaneous oxygen saturation (SpO_2_), noninvasive blood pressure (NBP), including mean arterial pressure (MAP), end-tidal carbon dioxide concentration in the expired air (ETCO_2_), and respiratory rate (RR). ETCO_2_ levels were measured using a gas-sampling nasal cannula. Prior to anesthesia induction, a peripheral venous catheter was inserted, and a physiological saline infusion was initiated. To measure and record patient state index (PSi) and electroencephalogram (EEG) continuously, we used the brain-function-monitoring module SedLine® (Masimo Corp., Irvine, CA, USA) and the patient monitoring platform Root® (Masimo Corp., Irvine, CA, USA). The SedLine® sensor was attached to the forehead, and the electrode impedance was checked. Modified Observer’s Assessment of Alertness/Sedation (MOAA/S), a validated scale for measuring the level of sedation (5 = complete alertness, 0 = completely unresponsive; Table 2), was used [10, 11].

**Table 2:** Modified Observer’s Assessment of Alertness/Sedation (MOAA/S) score

Score	Response
5	Responds readily to name spoken in normal tone
4	Lethargic response to name spoken in normal tone
3	Responds only after name is called loudly and/or repeatedly
2	Responds only after mild prodding or shaking
1	Responds only after painful trapezius squeeze
0	Does not respond to painful trapezius squeeze

**Fig. 2:**
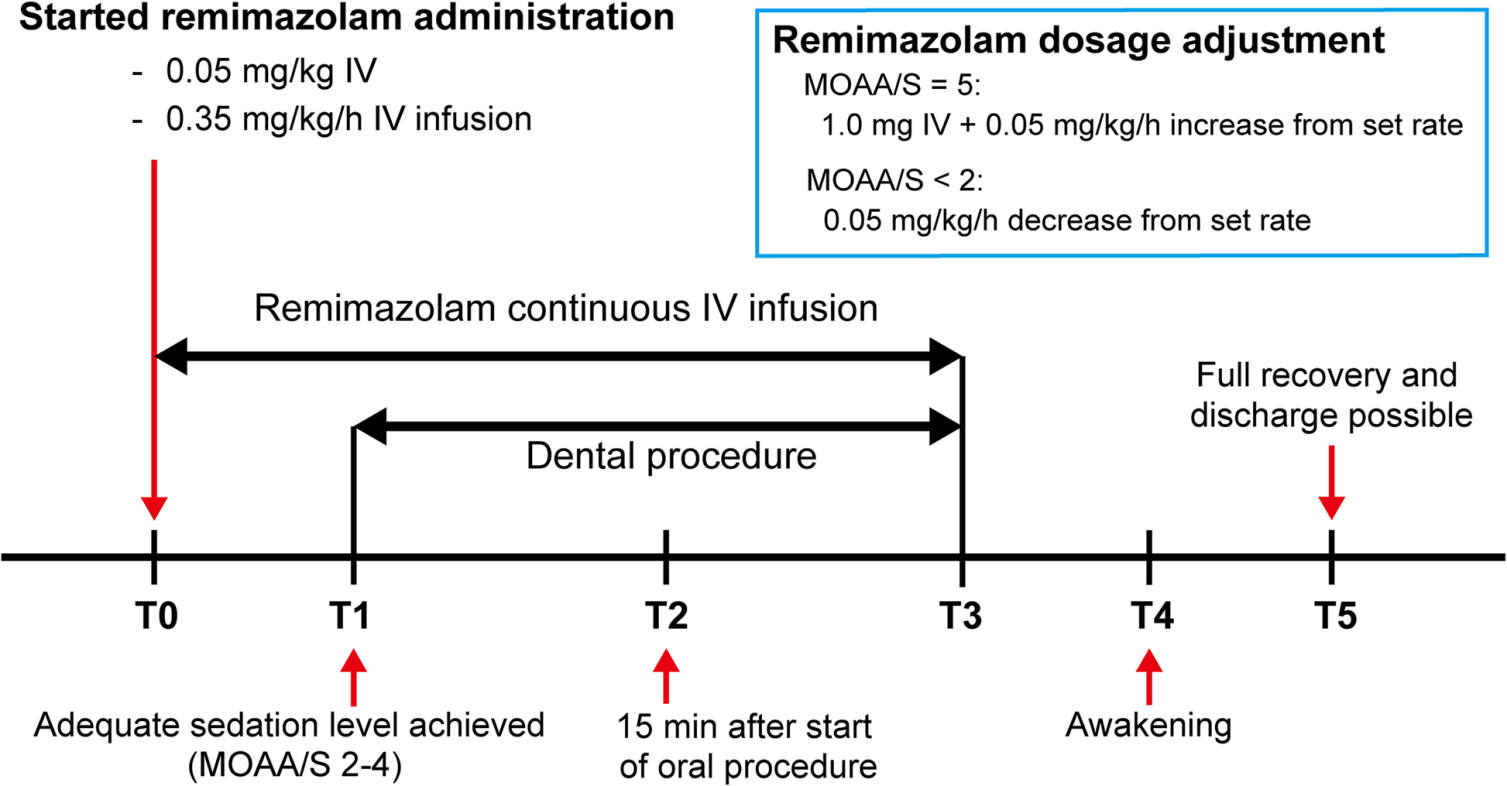
Study design of dental procedural sedation with remimazolam. T0: start of remimazolam administration, T1: adequate sedation level achieved, T2: 15 min after the start of the oral procedure, T3: end of the oral procedure and remimazolam administration, T4: awakening, T5: full recovery and discharge possible. IV: intravenous administration, MOAA/S: Modified Observer’s Assessment of Alertness/Sedation

The study design for sedation is shown in Fig. 2. Remimazolam (Anerem®, Mundipharma K.K., Tokyo, Japan) was administered as a single dose of 0.05 mg/kg followed by a continuous infusion at a rate of 0.35 mg/kg/h via a micropump. The infusion rate was adjusted to achieve the desired sedation level (MOAA/S score 2–4). The start of remimazolam administration was designated as T0. The point at which the adequate sedation level was achieved was defined as T1. If the target sedation level was not achieved, 1.0 mg of remimazolam was injected intravenously, followed by a 0.05 mg/kg/h increase in the infusion rate. The maximum dose rate of remimazolam was set at 0.6 mg/kg/h. If the target sedation level still could not be obtained, additional sedatives were used as rescue medications. When the sedation level deepened (MOAA/S score < 2), the continuous infusion dose rate was decreased by 0.05 mg/kg/h from the set rate. After achieving an adequate sedation level (MOAA/S score of 2–4), the oral surgeon administered local anesthesia and initiated the extraction of the third molar. The local anesthesia used was 2% lidocaine with adrenalin 1:80,000. The 15 min time point after the oral surgeon started the procedure was defined as T2. When the procedure was completed, the administration of remimazolam was discontinued (T3). Awakening was defined as the point at which the patient spontaneously opened his/her eyes (T4). If the patient did not awaken within 15 min after the end of administration, flumazenil, a benzodiazepine antagonist, was administered. Once the patient regained clear consciousness, the monitor was removed, and a coordinated movement test, the Romberg test, was conducted [12]. The Romberg test involved the patient standing upright with feet together and eyes closed. If the patient did not sway or topple for 30 s, they were discharged (T5). At the time of discharge, patients were asked to fill out a satisfaction survey and a memory questionnaire regarding the dental procedure. Patient satisfaction with the dental procedure was rated on a five-point scale: 1, very satisfied; 2, satisfied; 3, neutral; 4, unsatisfied; and 5, very unsatisfied. Patient memory of the dental procedure was also rated on a five-point scale: 1, none; 2, remembered a little; 3, remembered some; 4, remembered most; and 5, remembered all. The following day, patients were contacted to inquire about their condition and any adverse events experienced after leaving the treatment room.

### Study outcomes

The primary outcome of the study was the success rate of achieving an adequate sedation level with remimazolam monotherapy in extraction procedures. Secondary outcomes included induction time, awakening time, time until discharge, dosage of remimazolam, MOAA/S score, PSi, as well as the simulated plasma concentration (Cp) and simulated effect-site concentration (Ce) of remimazolam calculated from the recently reported Masui’s pharmacokinetic model [13, 14]. Other secondary outcomes consisted of patient satisfaction and memory of the procedure. Exploratory outcomes included adverse events, assessment of respiratory status (SpO_2_, ETCO_2_, RR, and upper airway stenosis or obstruction), assessment of circulation (HR, NBP, and ECG), delayed awakening from anesthesia, the need for oxygen therapy, and the need for airway management. Criteria for adjudicating adverse events included all unexpected clinical manifestations that occurred after the informed consent was signed. All adverse events were reported regardless of their relationship to the experimental drugs.

### Statistical analysis

All statistical analyses were conducted using the software package JMP® 16 (SAS Institute, Cary, NC, USA). Continuous variables were reported as mean and standard deviation (SD). The normality test feature in JMP® 16 was used for data analysis to determine whether the data followed a normal distribution. Normally distributed continuous variables were expressed as mean (95% confidence interval), whereas non-normally distributed continuous variables were expressed as median (interquartile range). Categorical variables were reported as absolute numbers and percentages. Pearson’s product rate correlation coefficient analysis was performed to assess the relationship between the MOAA/S score and PSi. Statistical significance was set at *p* < 0.05.

The sample size of 29 patients was estimated based on a threshold response rate of 75%, an expected response rate of 90% power, and an alpha value of 0.1 (one-sided), using the normal approximation. Accounting for potential dropouts, it was determined that 31 patients would need to be recruited.

## Results

### Primary outcome

The sedation success rate for achieving the desired sedation level with remimazolam monotherapy during the extraction procedure was found to be 100%.

### Secondary outcomes

The mean remimazolam induction dose was 0.08 mg/kg (95% confidence interval [CI], 0.07–0.09), and the mean time to achieve an adequate sedation level was 3.2 min (95% CI, 2.6–3.9). The mean infusion rate of remimazolam during the procedure was 0.40 mg/kg/h (95% CI, 0.38–0.42). The mean time from the end of remimazolam administration to awakening was 8.0 min (95% CI, 6.7–9.3), and the mean time from the end of remimazolam administration to discharge was 14.0 min (95% CI, 12.5–15.5). The mean MOAA/S score during the procedure was 3.7 (95% CI, 3.6–3.8), and the mean PSi during the procedure was 75.8 (95% CI, 72.2–79.4). Flumazenil was administered in one patient (3.4%) (Table 3).

**Table 3:** Primary and secondary outcomes

**Primary outcome**	
Sedation success	29 (100%)
**Secondary outcomes**	
Procedure time, min	37.5 (32.4–42.6)
Total duration of remimazolam infusion, min	42.6 (37.9–47.7)
Total dose of remimazolam, mg	19.6 (17.3–21.8)
Remimazolam induction dose, mg/kg	0.08 (0.07–0.09)
Time to achieve an adequate sedation level (MOAA/S score 2–4), min	3.2 (2.6–3.9)
Mean infusion rate of remimazolam during the procedure, mg/kg/h	0.40 (0.38–0.42)
Mean MOAA/S during the procedure	3.7 (3.6–3.8)
Mean PSi during the procedure	75.8 (72.2–79.4)
Time from the end of remimazolam administration to awakening, min	8.0 (6.7–9.3)
Time from the end of remimazolam administration to discharge, min	14.0 (12.5–15.5)
Use of flumazenil	1 (3.4%)

Variables are presented as mean (95% CI) or number of patients (%)

*Abbreviations: MOAA/S* Modified observer’s assessment of alertness/sedation, *PSi *Patient state index

### Effects of sedation on respiration and circulation

**Fig. 3:**
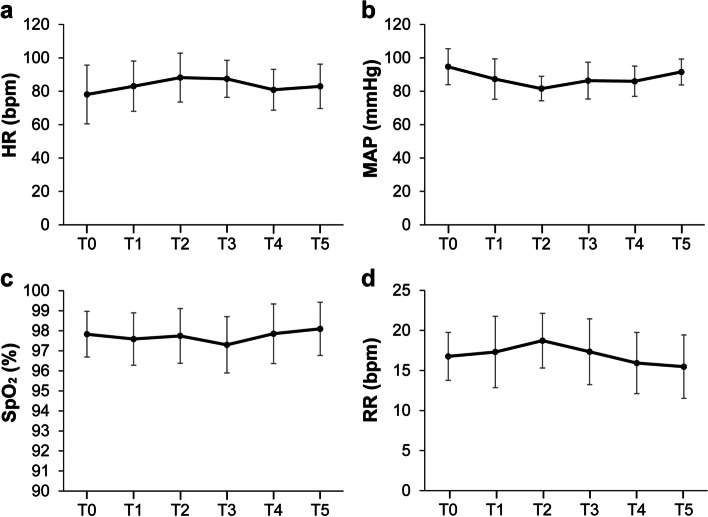
Change in vital signs against elapsed sedation time. HR: heart rate, MAP: mean arterial pressure, SpO_2_: percutaneous oxygen saturation, RR: respiratory rate. T0: start of remimazolam administration, T1: adequate sedation level achieved, T2: 15 min after the start of the oral procedure, T3: end of the oral procedure and remimazolam administration, T4: awakening, T5: full recovery and discharge possible. Results are expressed as the mean ± standard deviation

Figure 3 shows the trends of average HR, MAP, SpO_2_, and RR recorded during the sedation period. Additionally, Table 4 shows the effects of sedation with remimazolam on respiration and circulation. Although one patient had a decrease in respiratory rate (< 8 bpm), no significant respiratory or circulatory effects requiring intervention occurred during sedation. However, when water or saliva from the oral cavity entered the respiratory tract during the procedure, a coughing reflex was elicited in eight patients. All patients were able to expel the fluid by coughing, and these events did not result in decreased oxygen saturation.

**Table 4:** Effects of sedation on respiration and circulation

SpO_2_ < 90%	0 (0.0%)
Respiratory rate < 8 bpm	1 (3.4%)
Airway opening maneuver	0 (0.0%)
Oxygen therapy	0 (0.0%)
Facemask ventilation	0 (0.0%)
Cough reflex	8 (27.6%)
Injection pain	0 (0.0%)
HR < 50 bpm	0 (0.0%)
SBP < 80 mmHg	0 (0.0%)
ECG abnormality	0 (0.0%)

Variables are presented as number of patients (%)

*Abbreviations:ECG *Electrocardiogram, *HR* Heart rate, *SBP* Systolic blood pressure, *SpO*_*2*_ Peripheral oxygen saturation

### Depth of sedation level and EEG monitoring

**Fig. 4:**
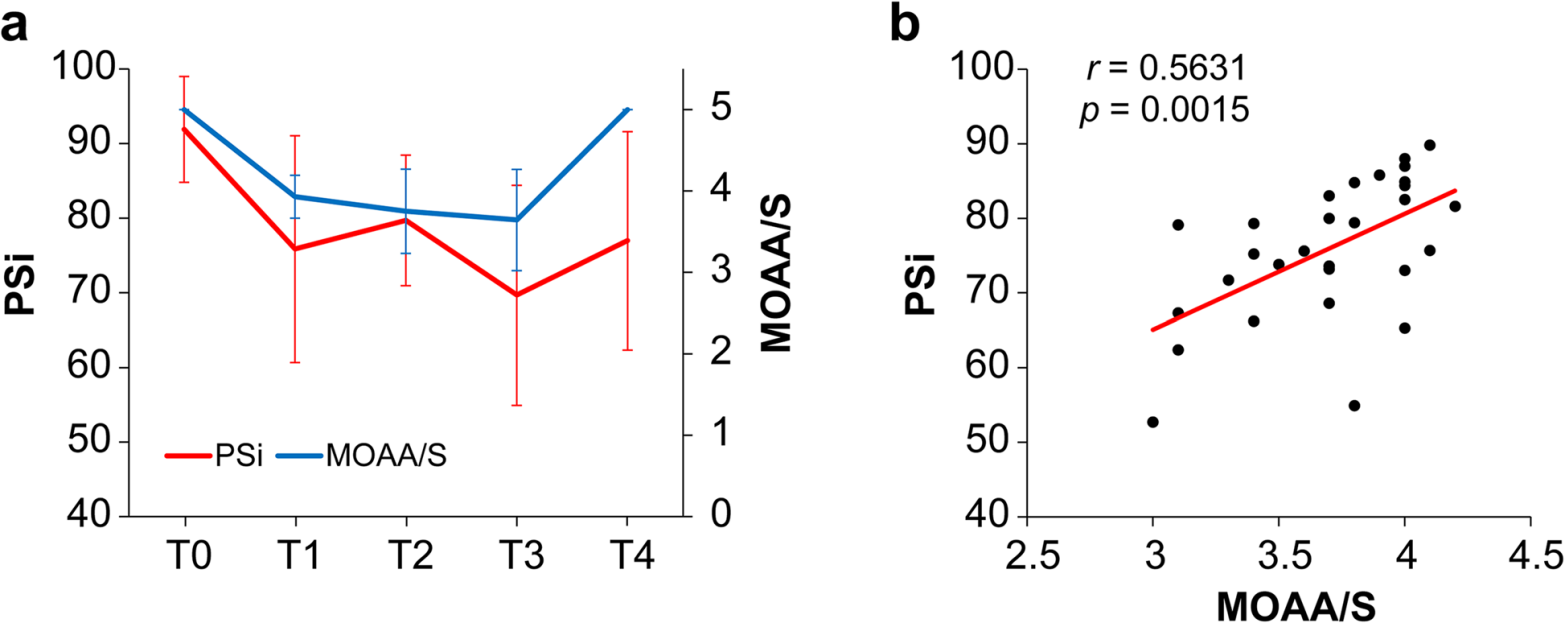
Relationship between Modified Observer’s Assessment of Alertness/Sedation (MOAA/S) score and patient state index (PSi) values. **a** Change in MOAA/S score and PSi values against elapsed sedation time. T0: start of remimazolam administration, T1: adequate sedation level achieved, T2: 15 min after the start of the oral procedure, T3: end of the oral procedure and remimazolam administration, T4: awakening. **b** Correlations between mean MOAA/S score and mean PSi values during the dental procedure. Results are expressed as the mean ± standard deviation. *r*: Pearson’s correlation coefficient, p: *p*-value for the statistical significance of the correlation

Figure 4a shows the changes in the MOAA/S score and PSi documented against elapsed sedation time. Changes in MOAA/S score and PSi were similar during the procedure. Figure 4b shows the correlation between the mean MOAA/S score and mean PSi during the procedure. A positive correlation was observed between the MOAA/S score and PSi (*r* = 0.5631, *p* = 0.0015).

### Adverse events post sedation

A few mild adverse events were reported after sedation. Specifically, one patient experienced a headache, another patient reported feeling unsteady after discharge, and one patient had malaise after discharge (Table 5). There were no cases of falls after discharge, and no instances of postoperative nausea and vomiting were reported.

**Table 5:** Adverse events after sedation

Unsteady after discharge	1 (3.4%)
Falls after discharge	0 (0.0%)
Headache	1 (3.4%)
Malaise	1 (3.4%)
Nausea	0 (0.0%)

Variables are presented as number of patients (%)

### Patient satisfaction and recall of procedure

The results of the satisfaction survey and memory questionnaires completed by the patients using five-point scales are shown in Table 6. The median total patient satisfaction score was 2 (interquartile range, 1–2). The percentage of patients who reported being satisfied or very satisfied was 89.7%. The median total patient memory of procedure score was 1 (interquartile range, 1–1). The percentage of patients who answered none or had little procedural memory was 96.6% (Table 6).

**Table 6:** Responses to the patient questionnaire

	1	2	3	4	5	Median
Patient satisfaction	13 (44.8%)	13 (44.8%)	2 (6.9%)	1 (3.4%)	0 (0.0%)	2 (1–2)
Memory of procedure	26 (89.7%)	2 (6.9%)	1 (3.4%)	0 (0.0%)	0 (0.0%)	1 (1–1)

Variables are presented as median (interquartile range) or number of patients (%)

Patient satisfaction: 1 = very satisfied, 2 = satisfied, 3 = neutral, 4 = unsatisfied, 5 = very unsatisfied

Memory of procedure: 1 = none, 2 = remember little, 3 = remember some, 4 = remember most, 5 = remember all

### Simulated Cp and Ce of remimazolam during the procedure

**Fig. 5:**
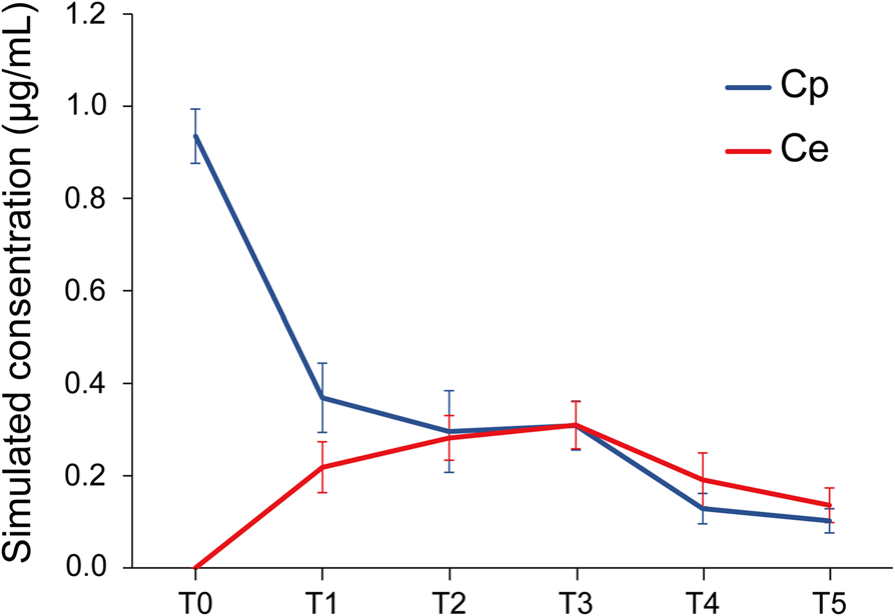
Change in simulated concentration of remimazolam against elapsed sedation time. Cp: plasma concentration, Ce: effect-site concentration. T0: start of remimazolam administration, T1: adequate sedation level achieved, T2: 15 min after the start of the oral procedure, T3: end of the oral procedure and remimazolam administration, T4: awakening, T5: full recovery and discharge possible. Results are expressed as the mean ± standard deviation

Figure 5 shows the changes in the mean simulated Cp and Ce of remimazolam documented against elapsed sedation time. The mean simulated Ce of remimazolam when an adequate sedation level (T1) was achieved was 0.22 µg/mL (95% CI, 0.20–0.24), the mean simulated Ce of remimazolam 15 min after the start of the oral procedure (T2) was 0.28 µg/mL (95% CI, 0.26–0.30), the mean simulated Ce of remimazolam at the awakening (T4) was 0.20 µg/mL (95% CI, 0.17–0.22), and the mean simulated Ce of remimazolam at full recovery and discharge (T5) was 0.14 µg/mL (95% CI, 0.13–0.16). The mean simulated Ce of remimazolam during the procedure was 0.29 (95% CI, 0.27–0.31) (Table 7).

**Table 7:** Simulated Cp and Ce of remimazolam during the procedure

Mean simulated Cp of remimazolam during the procedure, µg/mL	0.30 (0.28–0.32)
Mean simulated Ce of remimazolam during the procedure, µg/mL	0.29 (0.27–0.31)
Variables are presented as mean (95% CI)	

*Abbreviations: Ce* Effect-site concentration, *Cp* Plasma concentration

## Discussion

This study aimed to investigate the efficacy and safety of remimazolam for sedation in outpatients undergoing dental procedures and to determine the appropriate dosages of remimazolam. In our study, we found that remimazolam can achieve the depth of sedation required for impacted third molar extraction (without rescue sedation agents), rapid onset, and rapid recovery from sedation. In addition, sedation with remimazolam did not cause circulatory depression requiring intervention or respiratory depression requiring oxygen therapy, and no serious adverse events occurred.

Typical sedatives currently used for sedation in dental procedures include midazolam, propofol, and dexmedetomidine. Midazolam is a benzodiazepine with strong anxiolytic and amnesic effects, making it suitable as a sedative for patients undergoing dental procedures with fear or anxiety [15]. Compared to propofol, midazolam has been reported to cause less respiratory and circulatory depression [16]. Midazolam is metabolized by CYP3A4 [17], and its metabolism and elimination times are longer than those of propofol, resulting in a longer recovery time from sedation [18]. Flumazenil, a benzodiazepine antagonist, can be used to reverse the effects of midazolam if needed. However, overdosage of midazolam may lead to disinhibition [19] and re-sedation after antagonism [20]. Propofol has sedative and hypnotic effects via GABA receptors. Its high lipophilicity allows it to rapidly cross the blood-brain barrier, resulting in a rapid onset of action. It also has a short half-life and awakening time. However, propofol carries the risk of rapidly inducing deep sedation and causing respiratory and cardiovascular depression [21]. Therefore, physicians and dentists who administer sedation must possess adequate knowledge and skills pertaining to anesthesia and resuscitation [22]. In addition, propofol can cause pain upon injection [23], and there are no specific antagonists available for its reversal. Dexmedetomidine, an α2-receptor agonist with sedative, analgesic, and anxiolytic properties, is the first choice for sedation in the intensive care unit due to its minimal respiratory depression [24]. However, it requires an initial loading dose for administration, has a slow onset of action, and exhibits a long elimination half-life, thus, making it less commonly used for sedation in outpatients [25, 26].

Remimazolam, a new ultra-short-acting benzodiazepine anesthetic, has gained popularity in the use of procedural sedation and general anesthesia due to its favorable attributes, including rapid onset, short recovery time, and stable hemodynamics [27, 28]. Its metabolic pathway differs from that of midazolam since remimazolam is metabolized by hepatic tissue esterases. In a study examining the pharmacokinetics of continuous intravenous administration of remimazolam in 20 healthy patients, remimazolam showed high clearance (1.15 ± 0.12 L/min, mean ± SD), a small steady-state volume of distribution (35.4 ± 4.2 L), a short terminal half-life (70 ± 10 min), and a short context-sensitive half-life time (6.8 ± 2.4 min) [27]. Additionally, remimazolam-induced sedation can be reversed by flumazenil.

Several recent randomized controlled trials have compared remimazolam to propofol in procedural sedation such as gastric endoscopy and hysteroscopy. These studies have consistently demonstrated that the incidence of adverse events was significantly lower in patients administered remimazolam compared to propofol [29–31]. The time to loss of consciousness in the remimazolam group was longer compared to the propofol group [29]. Additionally, fewer patients in the remimazolam group reported injection pain compared to the propofol group [29, 30, 32]. Hemodynamic events and respiratory depression were also less frequent in the remimazolam group compared to the propofol group [29–32]. In a randomized controlled trial comparing remimazolam to midazolam, the success rates of the procedure were higher for remimazolam compared to midazolam. In addition, remimazolam had a shorter onset of action and faster neuropsychiatric recovery compared to midazolam [33, 34].

There are currently few research reports that have evaluated the efficacy and safety of remimazolam for sedation during dental procedures. A single-center, randomized, single-blind clinical trial with alfentanil in adults undergoing outpatient third molar extractions reported a lower incidence of adverse events, significantly lower incidence of injection pain, and significantly shorter recovery and discharge times in the remimazolam group compared to the propofol group [35]. Another prospective, randomized, controlled, single-center trial of 40 patients undergoing outpatient oral surgery in China found that the remimazolam group had a significantly higher sedation success rate and faster recovery compared to the midazolam group [36]. In a prospective randomized controlled trial of 83 patients with dental anxiety who underwent impacted tooth removal, the remimazolam group had significantly shorter onset, awakening, and recovery times, less frequent postoperative side effects, and higher patient and physician satisfaction than the midazolam group [37].

In our study, we observed a sedation success rate of 100% in achieving the desired sedation level with remimazolam monotherapy during dental extraction procedures. The time to achieve the target sedation level was 3.2 min (95% CI, 2.6–3.9), the awakening time was 8.0 min (95% CI, 6.7–9.3), and the time from the end of remimazolam administration to discharge was 14.0 min (95% CI, 12.5–15.5). Compared to the results of previous studies [33, 35, 38], sedation with continuous intravenous remimazolam administration was found to be suitable for sedation in outpatient dental procedures due to its early onset and rapid recovery of neuropsychiatric function. In addition, the effects of remimazolam sedation on patients’ blood pressure, HR, and SpO_2_ were minimal, and none of the patients required interventional treatment for respiration or circulation. Similar to previous studies [29–32], this study also found that remimazolam was safe to use in moderate sedation during dental procedures, without significant suppression of respiration or circulation.

During the dental procedures in our study, eight patients had a cough reflex due to water or saliva from their oral cavity entering the respiratory tract. Benzodiazepines have both peripheral and central muscle relaxant effects [39], and they also decrease the muscle tone in the upper airway [40]. Therefore, dental procedures during sedation with remimazolam may require consideration of the risk of aspiration due to water administration in the oral cavity. Additionally, one patient felt unsteady and was affected by postural imbalance after discharge. We performed the Romberg test to assess the recovery of neurologic function, but a more accurate method of evaluation may be needed to determine the recovery of neurologic function.

The post-treatment patient questionnaire revealed a high patient satisfaction, as well as an amnesic effect, strongly indicating the effect of benzodiazepines [39]. It has been reported that amnesia of procedures reduces anxiety and fear of dental treatment and has a positive effect on patient satisfaction [41]. Thus, the amnesic effect of remimazolam may reduce fear and anxiety of dental treatment.

Although previous studies have reported that a single intravenous dose of remimazolam can be used in some types of sedation, sedation with a single intravenous dose of ultrashort-acting remimazolam may not be sufficient to regulate sedation effectively in dental procedures, which typically require a longer duration of sedation compared to other procedures like gastrointestinal endoscopy [9]. Therefore, in this study, sedation was achieved through continuous intravenous administration of remimazolam. However, few reports exist on procedural sedation using continuous intravenous administration of remimazolam. As a mild-to-moderate level of sedation allowing for some degree of consciousness is deemed appropriate for dental procedures [41, 42], in this study, we determined the dose of remimazolam administration based on the findings of Schüttler et al., who reported that a remimazolam Ce of 0.34 µg/mL corresponded to a MOAA/S score of 4 (indicating a lethargic response of the patient to their name spoken in a normal tone) [27]. We adopted Masui’s pharmacokinetics model [13, 14] and set the initial remimazolam loading dose to 0.05 mg/kg, followed by an initial continuous infusion rate of 0.35 mg/kg/h, aiming to maintain a simulated remimazolam Ce of approximately 0.34 µg/mL during the maintenance phase of anesthesia. Based on the results of this study, an induction dose of 0.08 mg/kg, a continuous infusion rate of 0.4 mg/kg/h between procedures, and a simulated remimazolam Ce of 0.29 µg/mL appears to be suitable for maintaining a moderate level of sedation during dental procedures.

In this study, the level of sedation was assessed using both clinical assessment and EEG monitoring. The MOAA/S score was used to clinically assess the depth of sedation. In dental procedures, moderate sedation is often the desired level, where patients can respond purposefully to verbal commands, corresponding to a MOAA/S score of 3 [22, 41, 42]. Therefore, in this study, remimazolam dosing was adjusted to achieve a MOAA/S score of 2–4. Additionally, EEG monitoring was used to quantify and objectively evaluate the depth of sedation by analyzing the EEG signals. Previous studies have reported the usefulness of EEG monitoring in adjusting the depth of sedation during dental procedures [43–47]. However, processed EEG values (bispectral index value and PSi) were relatively higher during anesthesia with remimazolam compared to propofol [6, 48, 49]. The results of the current study showed a correlation between MOAA/S scores and PSi values in treated patients (Fig. 4b), and the PSi values were higher than those reported with propofol in another study [50]. These findings are consistent with the higher processed EEG values observed with the benzodiazepine remimazolam. Although further evaluation experiments are necessary, the PSi may be considered a useful indicator for assessing the patient’s sedation level during dental procedures with remimazolam.

Our study had some limitations. First, the study participants were on average relatively young adults, and only those without systemic complications were included. It remains unclear whether the procedure can be safely performed in pediatric patients, patients with cardiovascular or respiratory diseases, and older patients. The dosage and wake-up time of remimazolam may vary in such populations. Several clinical studies of short-term sedation with a single remimazolam dose in older adult patients have reported that it can be used safely with minimal respiratory and circulatory depression [32, 38, 51]. However, to the best of our knowledge, no studies of sedation with continuous remimazolam administration in children or older patients exist, and further research is needed. Second, the study only included the extraction of impacted third molars among various dental procedures. Third, the number of patients with severe gag reflexes was small, and it was not possible to assess whether remimazolam can suppress a severe gag reflex. Finally, this study was conducted at a single center without a comparative group, and the sample size was relatively small. Larger-scale, multi-center, randomized controlled trials are needed to validate and confirm the conclusions drawn from this study.

## Conclusions

In conclusion, the continuous administration of remimazolam demonstrates both safety and efficacy for sedation during dental procedures. It offers the advantages of an early onset of action and rapid recovery without suppressing circulation or respiration. Remimazolam proves to be a suitable sedative option for outpatient dental treatment. Furthermore, the results of this study also provided guidance on the appropriate dosage of remimazolam for achieving moderate sedation during dental procedures. In addition, the PSi value obtained from EEG monitoring using SedLine® might be a good indicator of the sedation level in dental procedural sedation with remimazolam.

## Data Availability

The datasets used and analyzed during the current study are available from the corresponding author upon reasonable request.

## Abbreviations

ASA: American Society of Anesthesiologists
BMI: Body mass index
Ce: Effect-site concentration
CI: Confidence interval
Cp: Plasma concentration
ECG: Electrocardiogram
EEG: Electroencephalogram
ETCO_2_: End-tidal carbon dioxide concentration in the expired air
HR: Heart rate
MAP: Mean arterial pressure
MOAA/S: Modified Observer’s Assessment of Alertness/Sedation
NBP: Noninvasive blood pressure
PSi: Patient state index
RR: Respiratory rate
SD: Standard deviation
SpO_2_: Percutaneous oxygen saturation
